# Keep It Simple–Diversity Strategy Takes Commitment and Accountability

**DOI:** 10.31486/toj.23.5037

**Published:** 2023

**Authors:** Deborah F. Grimes

**Affiliations:** Office of Diversity, Equity, and Inclusion, Ochsner Clinic Foundation, New Orleans, LA

Ochsner Health has a broad and bold vision of the future that will not only impact the health of citizens in the Gulf South region but also the face of health care delivery as a whole. Ochsner Health is committed to serving as the health care provider of choice in every region in which we have a presence and to being the employer of choice for workers in all segments of health care delivery. To successfully fulfill our vision, we must adopt a diversity and inclusion strategy that recognizes the evolving expectations of today's workforce and reflects the makeup of the populations we serve.

The health care industry is in the throes of unprecedented change.^1,2^ As older models of treatment, patient-provider relationships, employee compensation, and payment evolve, Ochsner Health has an opportunity to shape the landscape in unique and exciting ways. This changing health care terrain coincides with a significant increase in global interconnectedness, which has enhanced diversity in every sector of society. In the United States, nearly 15% of the population holds immigrant status, and, according to US Census data analyzed by The Brookings Institution, nearly 4 in 10 Americans identify with a race or ethnic group other than White^3^—a proportion that is likely to increase indefinitely into the future.

In seizing this moment, Ochsner Health can set the standard nationwide for the operation of an inclusive, equitable, and excellent workforce delivering culturally appropriate, tailored, superior health care.

The pursuit of diversity, equity, inclusion, and belonging at Ochsner Health will follow a phased approach. Each phase of the process will be informed and supported by rigorous data, evidence-driven practices, and comprehensive communication. The best available research in this area demonstrates that the most effective way to achieve diversity and inclusion is no different than the mechanism by which other organizational goals are realized: develop appropriate goals and metrics, share them with stakeholders, and embrace accountability for outcomes.^4^

Holistic cultural transformation, however, must serve as the ultimate aspiration and the means by which Ochsner Health leaves an indelible imprint on the practice of equitable health care. After the initial phase of establishing a foundation, Ochsner Health aims to move beyond a policy-based approach to these initiatives and toward the full integration of the ideals of true inclusion in all operations, employee expectations and practices, patient interactions, and communicative endeavors.

It is important to note that these phases are not discrete but will overlap and support one another, and in some cases, transformational inclusion will begin much earlier than anticipated. Generally, transformational inclusion is less a linear path than a progressive journey.

## PHASE ONE: ESTABLISHING THE FRAMEWORK, 2020-2024

The initial phase will continue to build upon the 5 pillars of the diversity and inclusion strategy already in place: care, leadership, environment, supplier diversity, and communication. Here are examples of actions taken in this initial phase:

1. Increase minority representation in senior leadership positions.
2. Decrease turnover of employees who belong to under-represented groups.
3. Adopt visible and accurate metrics for assessing the effectiveness of retention, promotion, mentorship, and training programs.
4. Equip leadership of all levels to lead, mentor, and empower diverse teams.
5. Establish relationships with diverse suppliers throughout the system and increase the number of minority-owned vendors utilized.
6. Increase cultural observances.
7. Increase employee resource group membership.

## PHASE TWO: OPERATIONAL INCLUSION TO TRANSFORMATIONAL INCLUSION, 2025-ONWARD

As a solid foundation for diversity and inclusion is embedded and initial training, education, and policy initiatives take root, inclusion will be actively and intentionally woven into leadership, operations, and behaviors throughout Ochsner Health. Ultimately, stakeholders become fully aligned with the goals of diversity, equity, inclusion, and belonging, and a culture of transformational inclusion is an integral part of all organizational actions.

An effective transition to phase two will be marked by 2 characteristics:

1. *A shift in advocacy toward leaders and frontline employees.* In phase one, the understanding is that staff members earmarked for diversity and inclusion will likely be its central advocates. In phase two, the chief advocates for establishing inclusion and transforming the culture at Ochsner Health should be its leaders, both on a system and a regional level, with the regional directors serving as support. Leaders at all levels are both the creators and guardians of organizational culture. The intention in phase one is to utilize education, training, mentorship, Ochsner resource groups, and communication to equip leaders to champion diversity and inclusion in their areas. Frontline employees will also become dedicated advocates of diversity and inclusion as the Ochsner resource groups increase their reach and impact.
2. *Integration of diversity and inclusion practices and frameworks into all operations, programs, and initiatives at Ochsner Health.* The natural outgrowth of ongoing conversations and collaborations across silos with an eye to diversity and inclusion will be full integration of diversity, inclusion, and equity principles into every aspect of Ochsner Health life. Rather than functioning as an addendum or amendment to business as usual, diversity and inclusion will share a symbiotic relationship with every other aspect and operation. Diversity and inclusion must be an intrinsic part of everything, from onboarding new employees to patient care to policy decisions to educational programs and everything in between. Lack of integration relegates diversity and inclusion to being an afterthought, a consideration tacked on after action is already decided on rather than a meaningful component to all operations. Transformational change only takes place when the new ideal becomes a nearly unconscious ethic. Conversations between the major stakeholders of all Ochsner Health operations and the diversity and inclusion personnel are the first step to determining best practices for integration. These conversations should include orientation and onboarding personnel, training and education, succession planning, advancement and promotion, team talent reviews, talent acquisition, workforce development, patient care, and marketing and communications. Based on these conversations, a joint plan of action can be developed to weave diversity and inclusion principles seamlessly and comprehensively into the workings of these areas.

Phase one initiatives will continue and expand as indicated, reinforcing needed cultural shifts.

## IMPLEMENT, MEASURE, and CREATE ACCOUNTABILITY

After integration of diversity and inclusion messaging and principles into other areas and operations within Ochsner Health, diversity and inclusion professionals will revisit these collaborative integrations with stakeholders to assess efficacy.

Organizations must address bias, prejudice, and historic inequalities as they embark on a health equity journey. It is important to identify important metrics to monitor success such as diversity hiring goals, tracking compliance with diversity educational training, inclusivity employee engagement scores, and diversity turnover metrics. These metrics should be attached to leader compensation and communicated in a transparent manner.

## References

[1] Figueroa CA, Harrison R, Chauhan A, Meyer L. Priorities and challenges for health leadership and workforce management globally: a rapid review. BMC Health Serv Res. 2019;19(1):239. doi: 10.1186/s12913-019-4080-7 31014349 PMC6480808

[2] Vallo Hult H, Hansson A, Svensson L, Gellerstedt M. Flipped healthcare for better or worse. Health Informatics J. 2019;25(3):587-597. doi: 10.1177/1460458219833099 30887867

[3] Frey WH. The nation is diversifying even faster than predicted, according to new census data. The Brookings Institution. Published July 1, 2020. Accessed August 8, 2023. brookings.edu/research/new-census-data-shows-the-nation-is-diversifying-even-faster-than-predicted/

[4] Hirsh E, Tomaskovic-Devey D. Metrics, accountability, and transparency: a simple recipe to increase diversity and reduce bias. Center for Employment Equity, University of Massachusetts Amherst. Accessed August 8, 2023. umass.edu/employmentequity/metrics-accountability-and-transparency-simple-recipe-increase-diversity-and-reduce-bias

